# Finite Element Models of Gold Nanoparticles and Their Suspensions for Photothermal Effect Calculation

**DOI:** 10.3390/bioengineering10020232

**Published:** 2023-02-09

**Authors:** José Manuel Terrés-Haro, Javier Monreal-Trigo, Andy Hernández-Montoto, Francisco Javier Ibáñez-Civera, Rafael Masot-Peris, Ramón Martínez-Máñez

**Affiliations:** 1 Instituto Interuniversitario de Investigación de Reconocimiento Molecular y Desarrollo Tecnológico (IDM), Universitat Politècnica de València, Camino de Vera s/n, 46022 Valencia, Spain; 2 Departamento de Electrónica, Universitat Politècnica de València, Camino de Vera s/n, 46022 Valencia, Spain; 3 Group of Electronic Development and Printed Sensors (ged+ps), Instituto Interuniversitario de Investigación de Reconocimiento Molecular y Desarrollo Tecnológico (IDM), Universitat Politècnica de València, AN34 Space, 7E Building, 46022 Valencia, Spain; 4 CIBER de Bioingeniería, Biomateriales y Nanomedicina (CIBER-BBN), Instituto de Salud Carlos III, 28029 Madrid, Spain; 5 Unidad Mixta UPV-CIPF de Investigación en Mecanismos de Enfermedades y Nanomedicina, Universitat Politècnica de València, Centro de Investigación Príncipe Felipe, 46012 Valencia, Spain; 6 Unidad Mixta de Investigación en Nanomedicina y Sensores, Universitat Politècnica de València, IIS La Fe, 46026 Valencia, Spain

**Keywords:** Finite Element Methods, metal nanoparticles, plasmonics, photothermal effect

## Abstract

(1) Background: The ability of metal nanoparticles to carry other molecules and their electromagnetic interactions can be used for localized drug release or to heat malignant tissue, as in the case of photothermal treatments. Plasmonics can be used to calculate their absorption and electric field enhancement, which can be further used to predict the outcome of photothermal experiments. In this study, we model the nanoparticle geometry in a Finite Element Model calculus environment to calculate the effects that occur as a response to placing it in an optical, electromagnetic field, and also a model of the experimental procedure to measure the temperature rise while irradiating a suspension of nanoparticles. (2) Methods: Finite Element Method numerical models using the COMSOL interface for geometry and mesh generation and iterative solving discretized Maxwell’s equations; (3) Results: Absorption and scattering cross-section spectrums were obtained for NanoRods and NanoStars, also varying their geometry as a parameter, along with electric field enhancement in their surroundings; temperature curves were calculated and measured as an outcome of the irradiation of different concentration suspensions; (4) Conclusions: The results obtained are comparable with the bibliography and experimental measurements.

## 1. Introduction

Noble metal nanoparticles (NPs) have a long history of usage, dating back to when they were formed as coatings for decorating glass. Today, NPs are used for various medical purposes, including drug delivery, medical imaging, diagnosis, and therapy. They can be passively or actively targeted to enter specific cancerous cells and can carry other molecules or use electromagnetic interactions for localized drug release or heating the tissue in photodynamic and photothermal therapies [1,2,3].

Nanoparticles can be heated using various methods, such as oscillating magnetic fields for iron oxide, radiofrequency fields for single-walled carbon nanotubes, and optical wavelengths for noble metal NPs ranging from two to hundreds of nanometers in diameter. The nanoparticle diameter and geometry can be adjusted to tune resonance in the absorption spectrum to the desired wavelength, enhancing light absorption and releasing energy as heat. The field of plasmonics, which provides analytical methods to calculate effects observed from the simplest nanoparticle geometries, grew out of classical electromagnetism. However, as more efficient geometries have been developed, classical approaches have become challenging to use, and simpler calculation methods are needed, such as numerical methods, which computers can handle easily [4,5,6,7,8,9].

The analytical models for the response of materials in an electromagnetic field are based on Maxwell’s electromagnetic theory and treat matter as a set of charged particles. The electric and magnetic fields of the incident wave cause the electrons of the material’s atoms to oscillate [10,11]. If a particle is smaller than the wavelength of the electromagnetic field, like a nanoparticle, the oscillation is confined to a small volume, making the effects different from those of the bulk material. For nanoparticles much smaller than the wavelength, the analysis can be simplified by assuming the same wave phase on each oscillation, making it based on an electrostatic field, which works well for particles smaller than 100 nm [12].

The analysis of Maxwell’s equations for nanoparticles shows two key conclusions [13]. One, the nanoparticle’s polarizability displays a resonant enlargement at a specific wavelength due to confinement of the plasmon, known as Localized Surface Plasmon Resonance (LSPR), which varies based on the nanoparticle’s geometry, material, and surrounding medium and is exploited in most of their applications. Two, the resonant polarizability also results in an enhancement of the electric field at the exterior of the nanoparticle, making the nanoparticles useful in optical devices that sense changes in the electric field [14,15,16].

The absorption and scattering cross-sections of nanoparticles are affected by the LSPR, which enhances them at the resonant wavelength. Noble metal nanoparticles, like gold, typically exhibit enhanced absorption, peaking in the Near-Infrared (NIR) due to their atomic properties [17]. The LSPR is influenced by the shape, size, material, layers, and coatings of the nanoparticle, as well as the medium it is suspended in, contributing to the dielectric function and polarizability [18,19]. Hence, adjusting these parameters allows a nanoparticle to be designed to absorb mainly in the NIR region [20,21]. The theoretical framework of this study is described in detail in Supporting Information.

Numerical methods, such as Finite-Difference Time-Domain (FDTD) and Finite Element Method (FEM), and boundary methods like Multiple Multipole Program (MMP), Method of Auxiliary Sources (MAS), Discrete Dipole Approximation (DDA), Boundary Element Method (BEM) and Meshless Boundary Integral Equation (MBIE) are commonly used for custom and complex geometries [22,23]. Deep Neural Networks have been employed to improve computation efficiency by using Machine Learning algorithms with comparable accuracy [24].

The FDTD and FEM are the most widely used numerical methods for analyzing electromagnetic problems. The FDTD method discretizes the physical space into Yee cells, where the electric and magnetic fields are calculated and updated until convergence is reached. The FEM solves Maxwell’s equations by discretizing the geometry and approximating the fields. Both methods can become problematic with dense meshes [25].

The FDTD method has been used for the simulation of near-field and far-field optical properties [26,27], solar cell efficiency enhancement [28,29], and surface-enhanced Raman scattering analysis [30,31]. On the other hand, FEM is used for the simulation of near-field and far-field optical properties of geometrically complex architectures without the need for the previous simplification [32,33], the simulation of fluorescence [34,35], and bio-related applications [36,37], such as photothermal therapy [38,39], among other applications [40,41].

Gold nanoparticles have a pronounced LSPR, allowing for efficient NIR light absorption and thermal energy conversion. Gold NanoStars (AuNSts) have improved light-to-heat conversion due to strong electromagnetic field amplification at sharp tips. They have good stability, biocompatibility, and can be easily functionalized and incorporated into hybrid nanoparticles [42,43,44]. However, noble metals have a high free electron density causing excessive resistive loss and limited plasmonic tunability. Moreover, the materials needed for their syntheses are expensive. Noble metal-free alternatives, such as semiconductor nanocrystals (WO3-x, Cu2-xS, Cu2-xSe), exhibit LSPR absorption in longer wavelength regions and are finding their field of applicability in solar energy applications [45,46,47].

This study uses the FEM to calculate the response of gold NanoRods and NanoStars to an electric field simulating laser irradiance. The effects of varying the geometry on the LSPR location are calculated and compared with experimental measurements. The study also uses the gold NanoStar results to develop a model for a set of NPs suspended in distilled water, simulating the temperature rise produced by enhanced absorption during irradiation. This model is adjusted and validated by experimental temperature measurements. With this, we aim to make a step towards developing a set of methods to plan ahead the outcomes of a photothermal treatment, even from the design and optimization of the nanoparticles.

## 2. Materials and Methods

### 2.1. Nanoparticle EM Model

First, modeling was carried out for one of the simplest, non-toxic particles used experimentally, golden NanoRods, inspired by previous data [48]. The objective was to calculate the absorption and scattering cross-section and the electric field enhancement for one NanoRod in the near-visible and visible spectrum of frequencies.

The development of the model started with a series of steps that are common to this, and the rest of the models are described further in the text, which is the preparation of the COMSOL interface, setting up the project, and setting of the boundary geometries. The simulation was carried out in COMSOL 5.5, in a machine featuring an AMD Ryzen Threadripper 3960X 24-core processor and 128 GB RAM, with Windows 10 Enterprise as the operating system. The Radio Frequency module was used, as it contains the tools for solving Maxwell’s equations, the Drude model, Mie solutions, etc., as required. The analysis was carried out in the frequency domain.

Next, the particle parameters were defined: this is particularly useful if the analysis is parametric; for example, the different absorption cross sections spectrums could be calculated using the parametric variation of radius, longitude, and medium refractive index.

**Figure 1:**
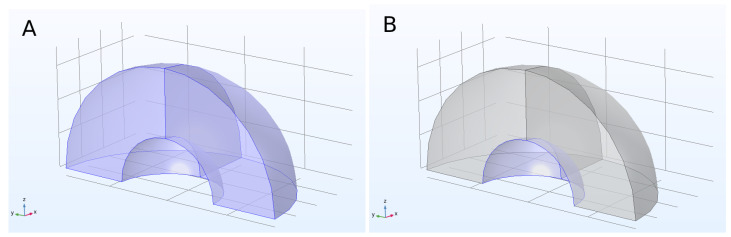
(**A**) A quarter of the PML domain and (**B**) a quarter of the scattering boundary are highlighted.

First, a NanoRod of radius r0 of 6 nm was defined. Its length was given by two times the radius times the aspect ratio a_r, which was defined as 3.5 (arbitrary number), giving an l_gnr length of 42 nm (tip to tip) for this first calculation. Other important parameters to define were the scattering boundary radius, which was set to 150 nm, and the Perfectly Matched Layer (PML) domain radius of 200 nm. The PML is an artificial absorbing layer that truncates computational regions in numerical methods to simulate problems with open boundaries. In this layer, the waves are completely absorbed to avoid them returning as reflections; this sets one of the boundary conditions for the solution of the model. Contrary to this, the scattering boundary marks the borders of the space where the medium and particle are situated and defines the region where the light extinction is calculated. Both PML and scattering boundary regions were configured as shown in Figure 1A and Figure 1B, respectively.

Finally, the refractive index of the medium was set to a constant 1.33, the standard value for water; and the incident irradiance power $I_{np}$ was set to 1 W/cm${}^{2}$. The wavelength of the irradiance was set to 532 nm, but this is not relevant as the wavelength is the parameter to change to achieve the calculation in the wavelength spectrum. The parameters introduced are summarized in Table 1.

For the next step, the geometry of the NanoRod was defined using two spheres of radius r0 on the ends of a cylinder of radius r0 and length l_gnr-r0*2 (so the total length adding the spheres is l_gnr), enabling the parametric geometry variation, and then joining the three parts in one domain that was called np, deleting the parts of the spheres that fell inside the cylinder.

As the geometry of this study was symmetrical, the calculations could be carried out for just a quarter of the whole geometry, then multiplying the results by 4 to obtain the total. For this purpose, the geometry was cut, leaving only the first quarter of the sphere and nanoparticle.

For the plane wave simulation, the software needs to add a Perfect Electrical Conductor (PEC) and a Perfect Magnetic Conductor (PMC) plane. As the wave was set to travel in the positive y direction, the x-y plane was defined to be the PEC plane and the y-z plane the PMC plane.

To add the material properties, the COMSOL Material Library was used. H${}_{2}$O was defined for the surrounding medium and gold for the nanoparticle. The gold definition uses the parameters set by Rakić et al., which were fit from multiple experimental data and are suitable for this model [49].

In the next steps, the physics were set for the study. The model was solved for the scattered field, so a background electric field ($E_{0}$) was added. It was defined to travel in the y direction, so the electric field was polarized along the *z*-axis, which was the longest axis of the particle. This is important as the polarization of the particles is different as their position with respect to the electric field changes. The electric field is defined as (1).

(1) $$E_{0}=\sqrt{\frac{2\cdot I_{np}}{c\cdot \varepsilon_{0}\cdot \sqrt{\varepsilon_{r_{av}}}}}$$

where *c* is the speed of light, $\varepsilon_{0}$ the vacuum permittivity, and $\varepsilon_{r_{av}}$ the average relative permittivity for the chosen materials. To direct the wave in the negative *y*-axis direction, the $E_{b}$ vector was set as (2).

(2) $$E_{b}=E_{0}e^{-i\kappa y}\;\;\;\;\left(V/m\right)$$

where κ is the wave propagation constant for the materials.

Once the model was set, the mesh which defines the finite element sizes was created. As a general rule, the mesh size must be smaller than λ/8, which affects the space of the medium and boundaries, and six times smaller than the smallest dimension of the particle, which in this case was r0/6, to obtain enough elements to represent its geometry correctly. The PML domain meshed as an even distribution of five layers since its resolution is not a factor in the results.

As a result of the calculation of the model, the following variables are found:

- $Q_{h}$, the cycle averaged total power dissipation density by resistive loss in ${W/m}^{3}$
- $P_{o_{av}}$, the time average Poynting vector components of the scattered field in ${W/m}^{3}$
- $E_{norm}$, the amplitude of the electric field calculated as $E_{norm}=\sqrt{E_{x}^{2}+E_{y}^{2}+E_{z}^{2}}$ in V/m

To find the total power dissipation of all the elements in the nanoparticle, which corresponds to the resistive loss of absorbed light, an integration (3) over the nanoparticle (np) volume (V) was carried out.

(3) $$W_{abs}={\;\int \int \int \;}_{V_{np}}Q_{h}dV\;\;\;\;\left(W\right)$$

Similarly, to find the total power scattered, the integration (4) of the Poynting vector was carried out on the surface of the imaginary sphere around the nanoparticle, which was defined as the scattering domain. *n* is the unit vector normal to the sphere.

(4) $$W_{sca}={∯}_{S}(P_{o_{av}}\cdot n)dS\;\;\;\;\left(W\right)$$

These results are still in a form that depends on the irradiated power $I_{np}$, and two other operations must be carried out to find the required characteristics of the model. As a quick reminder, this result is characteristic of the model broadly, as it depends on the medium refractive index, the materials, and the particle geometry. Finally, the operation was multiplied by 4 to calculate the result in the whole geometry, as it was cut into a quarter to simplify the problem thanks to symmetry.

Next, the absorption cross-section of the nanoparticle was calculated as in (5).

(5) $$\sigma_{abs}=4\cdot W_{abs}/I_{np}\;\;\;\;\left(m^{2}\right)$$

Similarly, the scattering cross-section of the nanoparticle in (6).

(6) $$\sigma_{sca}=4\cdot W_{sca}/I_{np}\;\;\;\;\left(m^{2}\right)$$

These results were calculated for each wavelength introduced in the parametric analysis. Furthermore, a set of calculations were carried out, introducing the aspect ratio as a parameter, varying it between 1.5 and 5. The relative tolerance of the iterative solver was set to 0.01.

### 2.2. Gold NanoStar Model

The next model developed represents the nanoparticles used in the experimental section. To this end, images were taken with electron microscopy, and the core, spike bases, and tips were measured, resulting in an average core size of 12.5 nm, and spikes around 8 nm in length and 3 nm radius. The core was modeled as a sphere and was surrounded by cylindrical spikes with spherical ends. As it was carried out with the NanoRod, at least one of the spikes was situated fully transversely to the electric field to measure the maximum absorbance efficiency, and more spikes were added in 45${}^{\circ }$ spacings around the sphere. The spikes parallel to the electric field were omitted once the first calculation was done as they did not affect the result but significantly lengthened the calculation time.

The calculation took advantage of symmetry to calculate only one quarter of the geometry, with an 8 nm spike length and varying the spike length as a parameter, which resulted in different absorption cross-section spectrums. The calculated results were compared with the absorption spectrums of three NanoStar syntheses that resulted in morphologies with different tip lengths.

### 2.3. NanoStar Suspension in a Well Model

The last model that was developed represents the suspension of gold NanoStars in water. As the NanoStars are so small, they were considered punctual heat sources dependent on the incident laser power. They were suspended in distilled water in concentrations typical of experimental applications, so the model was reduced to a cylindrical geometry, divided into finite elements which contained several nanoparticles; therefore, each element was heated in the function of the number of nanoparticles and the laser power that reached it. The geometry representing the water was surrounded by a plastic cylinder, open at the top, simulating the well of a 96-well plate.

Previously, the laser-irradiated area was much larger than the nanoparticle, but now, it only irradiated a portion of the well. To simulate this, the irradiated area was implemented, along with a Gaussian function that made the irradiation power greater in the center and lower closer to the area limit. Moreover, the Beer-Lambert law was implemented in the function of the nanoparticle absorption cross section and concentration to correctly simulate the attenuation of the laser beam through the suspension.

First, attenuation of the suspension was calculated as in (7) by the Beer–Lambert law using the absorption cross-section and concentration, per unit volume, where *C* is the concentration in mmol and $N_{A}$ the Avogadro constant in mol${}^{-1}$.

(7) $$\mu =\sigma_{abs}\cdot C\cdot N_{A}/10^{3}\;\;\;\;\left(1/m\right)$$

In (8), the output power $\left(P_{0}\right)$ of a 500 mW, 808 nm laser was distributed first in a circle to give approximately a 4 W/cm${}^{2}$ irradiance power, as in the actual application with a radius $r_{l}$ of 2 mm.

(8) $$I_{0}=\frac{P_{0}}{\pi r_{l}^{2}}\;\;\;\;\left({W/m}^{2}\right)$$

Then, a two-dimension normal distribution was applied to the power by the (9) function where $\sigma_{n}$ is the standard deviation and is set to $\sigma_{n}=2\;\;\left({\mathrm{mm}}^{2}\right)$.

(9) $$I=I_{0}\cdot e^{-{\left(\frac{x^{2}+y^{2}}{\sigma_{n}}\right)}^{2}}\;\;\;\;\left({W/m}^{2}\right)$$

The distribution of power in the simulation was first validated by calculating the irradiation at the center of the suspension and at depth z, and then in a perpendicularly traced line in the *x*-axis at different depths.

This power was absorbed through the suspension following the Beer–Lambert law such that the power that was sourced as heat in a volume situated at a z depth below the surface (and displaced from the center in x-y) was calculated by (10).

(10) $$Q_{0}=\mu \cdot I\cdot e^{-\mu z}\;\;\;\;\left({W/m}^{3}\right)$$

**Figure 2:**
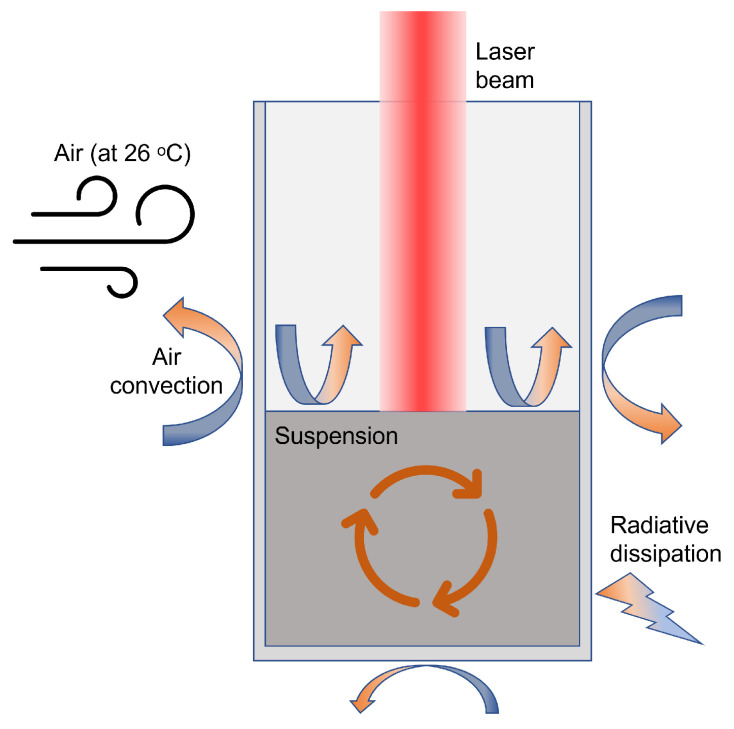
Heat dissipation methods implemented in the model.

Dissipation methods were implemented as shown in Figure 2 to avoid a result with infinitely increasing temperature. First, water convection was implemented, a method that COMSOL handles natively, combining all elements of the water geometry. Second, heat radiation and conduction to plastic and then air were implemented. Air is considered to be infinitely large and with convection, so its temperature never alters.

Dissipation to air by convection requires a heat transfer coefficient $h_{c}$, which was experimentally adjusted inside a range of normal values, in the case of water to air and plastic to air, 10 to 500 W/m${}^{2}$K, considering the experiment was carried out in a room with cold air conditioning at 26 °C ambient temperature.

### 2.4. Experiments on Suspensions of Gold NanoStars

To calibrate and validate the model, various suspensions of gold NanoStars in water were irradiated, and the sample temperature was measured.

**Figure 3:**
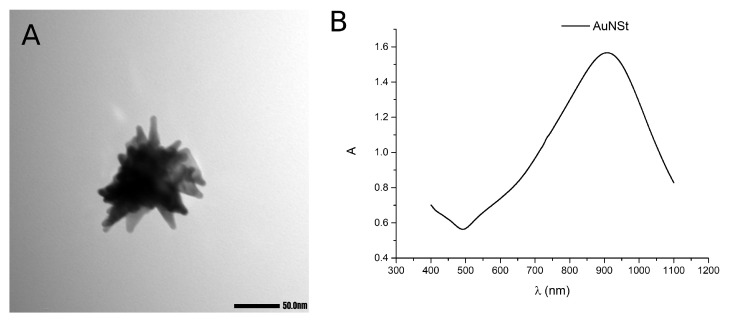
(**A**) Morphology of the gold NanoStar synthesis under electronic microscopy, and (**B**) measured absorption spectrum. A.U. normalized at 400 nm (ε = 2400 L mol${}^{-1}$cm${}^{-1}$ for Au atoms).

The NanoStars were synthesized following previously described methods [42], then characterized by their absorption spectrum and measured with electron microscopy, where some images of their morphology were taken, as seen in Figure 3.

The experiment was carried out by suspending gold NanoStars in 100 μg/mL, 50 μg/mL, and 25 μg/mL concentrations, then putting 200 μL of these suspensions in 96-well plates wells and irradiating them consecutively at 4 W/cm${}^{2}$ and 808 nm for 5, 10, and 15 min. The experiments were performed twice for the 100 and 50 μg/mL concentrations. While irradiating, the sample temperature was measured with an AMG8833 thermographic camera module, previously calibrated with a Testo 875 thermographic camera. From the camera measurements, delivering an 8 × 8 pixel matrix each second, the hottest spot was used to create the temperature evolution curves.

To compare the model with the experimental results, the absorption cross-section of the suspension model was set to 3.6322 × 10${}^{-15}$ m${}^{2}$, the same as the individual nanoparticle absorption cross-section calculation result. The model was calibrated with preliminary results of a 100 μg/mL, 200 μL suspension of the same gold NanoStars, setting the suspension to air heat transfer coefficient to $95\;\;W/(m^{2}K)$, and the well to air heat transfer coefficient to $40\;\;W/(m^{2}K)$. Once the calibration was done, the concentration was set to 50 μg/mL and 25 μg/mL, and the temperature calculated on the center of the surface of the suspension to compare the result with their respective experimental curves.

## 3. Results and Discussion

### 3.1. Modeled Geometries

**Figure 4:**
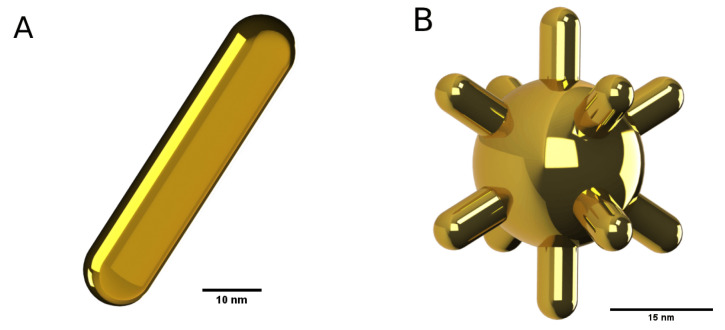
(**A**) Aspect of the NanoRod and (**B**) NanoStar geometries once built.

For reference, the geometries of the models of the gold NanoRod and NanoStar can be seen in Figure 4.

### 3.2. Absorption and Scattering Cross-Section Results

#### 3.2.1. NanoRod Results

**Figure 5:**
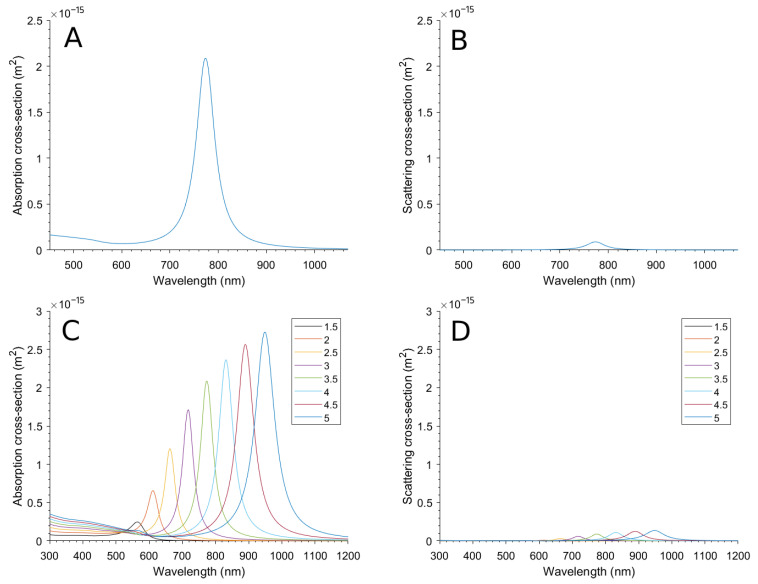
(**A**) Absorption cross-section and (**B**) scattering cross-section of the NanoRod with an aspect ratio fixed to 3.5. (**C**) Absorption cross-section and (**D**) scattering cross-section of the NanoRod when the aspect ratio is varied between 1.5 and 5.

The single aspect ratio NanoRod calculation results in the absorption and scattering cross-section spectrums of Figure 5A and Figure 5B, respectively. On these figures, the calculated magnitude varies with the wavelength of the incident electromagnetic fields, and a peak due to the LSPR can be observed. Here, the peak absorption occurs at 778 nm for a 3.5 aspect ratio.

The multiple calculations with different aspect ratios can be seen in Figure 5C,D. It can be observed that the LSPR wavelength red-shifts when making the aspect ratio larger, which is due to the distances between charges in the dipole being enlarged, so the damping forces are less effective [50,51]. The scattering is almost negligible compared to the absorption.

#### 3.2.2. NanoStar Results

**Figure 6:**
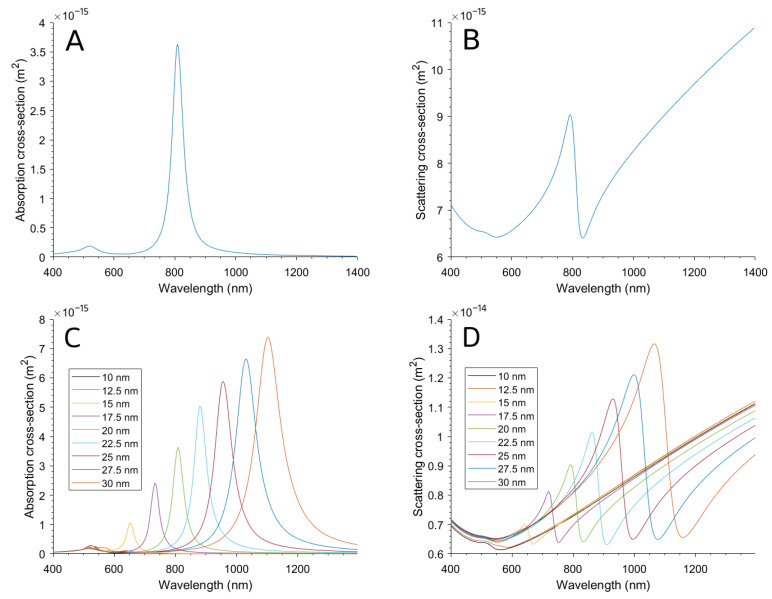
(**A**) Absorption and (**B**) scattering cross-sections of the NanoStar with tip length fixed to 20 nm. (**C**) Absorption and (**D**) scattering cross-sections varying tip lengths between 10 and 30 nm.

In the same way as before, the NanoStar absorption and scattering cross-section calculation results with a single tip length can be seen in Figure 6A,B. In this case, the scattering cross-section is larger, which could be due to the presence of the nucleus and more tips and their interactions.

As the resulting spectrum peaks at 809 nm, close to the 808 nm wavelength of the application laser, the result of peak 3.6322 × 10${}^{-15}$$m^{2}$ absorption cross-section is noted to use in the NanoStar suspension in a well model. This peak is larger than the previous result from the NanoRod; moreover, the NanoStar has multiple tips, so the probability of having one or more tips perpendicular to the electric field is greater, so their absorption efficiency in experimental applications is better.

**Figure 7:**
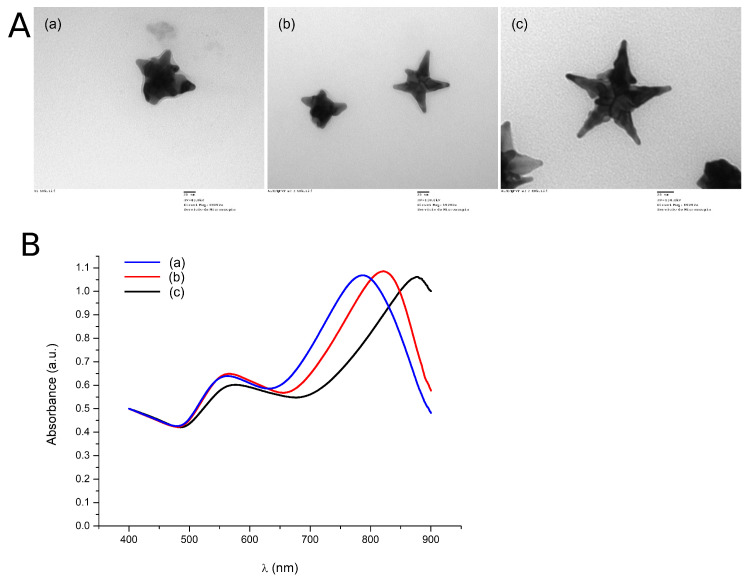
(**A**) NanoStar morphologies from three different syntheses with different tip lengths from shorter (a) to longer (c) and (**B**) Measured absorption spectrums corresponding to the synthesis of the NanoStars. A.U. normalized at 400 nm (ε = 2400 L mol${}^{-1}$cm${}^{-1}$ for Au atoms).

The calculations varying the tip length yielded the results summarized in Figure 6C,D. The spectrum can be seen to red-shift when making the tips longer. This red-shifting was corroborated by the characterization of three NanoStar syntheses that resulted in morphologies with different tip lengths, as seen in Figure 7A, and correspond to the absorption spectrum seen in Figure 7B, also red-shifting for longer nanoparticle tips.

### 3.3. Electric Field Enhancement Results

**Figure 8:**
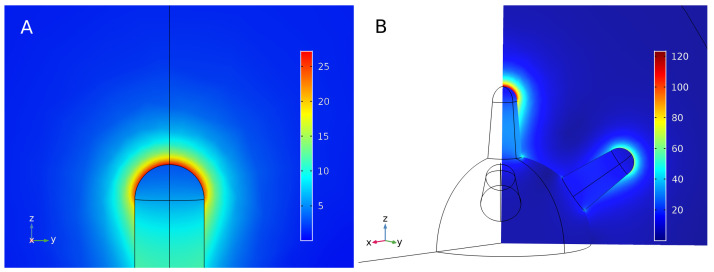
(**A**) Enhancement of the electric field on one tip of a NanoRod with aspect ratio 3.5 and situated in a 772 nm wavelength electromagnetic field and (**B**) of a NanoStar with 20 mm tip length situated in an 809 nm wavelength electromagnetic field.

Concerning the electric field enhancement of the NanoRod, shown in Figure 8A, the maximum is an enhancement of 27.21 when the wavelength is 778 nm and close to the tip of the NanoRod. The field enhancement is greater when close to the nanoparticle, as predicted by the analytical models, and decreases rapidly when moving away. The calculation of the electric field enhancement in the proximity of the nanoparticle might be of use in the future to calculate electromagnetic interactions with other nearby particles or molecules, such as two-photon activation or electromagnetic release of drugs.

In a NanoStar, the electric field enhancement can be observed on the various tips of the nanoparticle. For this purpose, a plane was set to observe the main tip, fully transversal to the electric field, and one of the tips at 45${}^{\circ }$, in Figure 8B. In this case, the electric field enhancement achieved 123.4 times the magnitude of the original field near the main tip and 75 times near the 45${}^{\circ }$ tips, which is greater than that of the NanoRod, making the NanoStar more suitable for applications relying on electric field enhancement.

### 3.4. NanoStar Suspension in a Well Model

**Figure 9:**
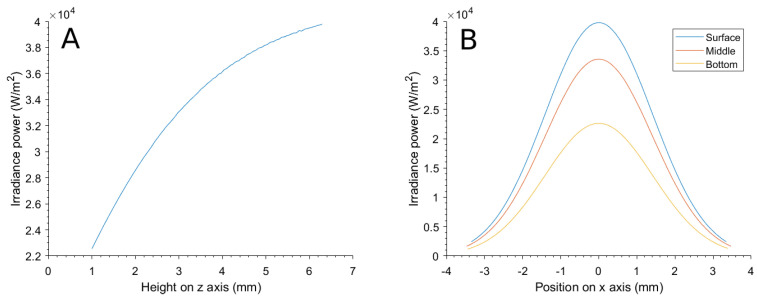
(**A**) Distribution of the laser power over a *z*-axis line through the center of the suspension and (**B**) over an *x*-axis line at different z depths.

The calculation of the effects of the implementation of the attenuation and distribution of the laser power delivered the results seen in Figure 9.

The *z*-axis distribution in Figure 9A shows that the irradiation power is the maximum at the surface (point of maximum height from the coordinate origin) and center of the suspension and decreases with depth, complying with Beer–Lambert’s law. The *x*-axis line calculation in Figure 9B shows that the normal distribution of the power in the surface is correctly implemented, and at different depths, the distribution is also affected by the attenuation.

**Figure 10:**
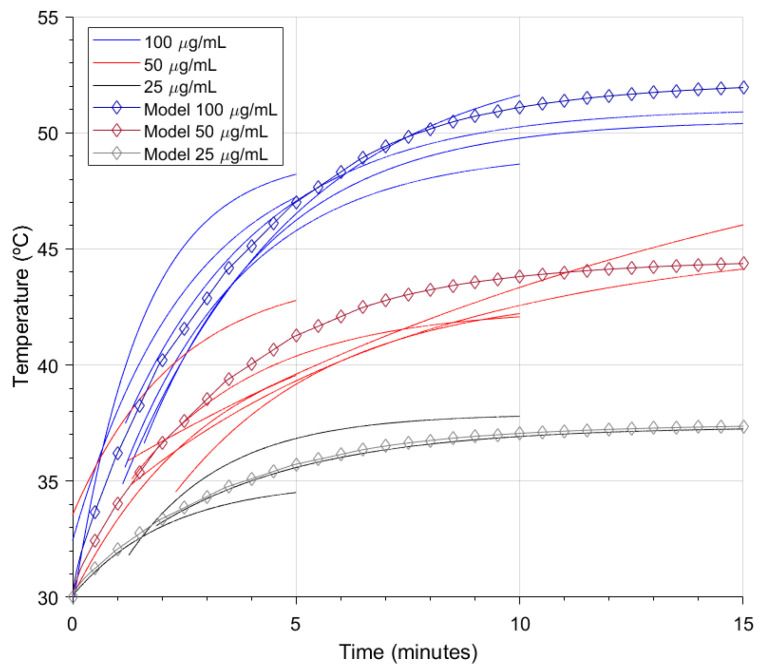
Temperature evolution in the model and experimental measurements when irradiating various suspension concentrations at 4 W/cm${}^{2}$ 808 nm.

These results report that the power attenuation and distribution were correctly implemented. Next, the temperatures that resulted from the calculations were plotted against experimental temperature curves as seen in Figure 10.

Compared to experimental measurements, the model could predict the experiment’s outcomes by approximating the temperature curve. The model could only calculate heating by individual photothermal effects of the nanoparticles in the suspension. However, as the experimental concentration was low, the collective effects were considered negligible. Due to computing power limitations, the model was constructed as a macroscopic simplification where heat is transported by diffusive mechanisms, but ballistic thermal transport should be considered when modeling at the nanoscale, as heat is transported differently from the nanoparticle to the medium [52,53].

## 4. Conclusions

In conclusion, the Finite Element Method (FEM) was used to model and analyze the properties of NanoRods and NanoStars, as well as a suspension of NanoStars. FEM had a major advantage which was the relatively easy implementation of a model of a complex nanoparticle morphology, at the cost of its major disadvantage, the computation cost of an elevated number of elements. Nevertheless, the available technology provided the results in 58 min for the NanoRod and 32 h for the NanoStar. The calculations of absorbance and scattering cross-section, as well as electric field enhancement, showed good agreement with experimental results for the individual nanoparticle models. A key contribution of this work was the development of the FEM for the suspension of NanoStars, which was based on the data from the individual nanoparticle model and used to predict the temperature evolution when irradiated with an 808 nm laser. These results have important implications for photothermal therapy, as they provide a better understanding of the behavior of NanoStars in suspension and how they can be used to generate heat for therapeutic purposes. Further work could focus on developing more sophisticated models to better understand the interactions of nanoparticles with living tissue.

## Figures and Tables

**Table 1:** Summary of the parameters introduced for the NanoRod model.

Name	Expression	Units	Description
r0	6	nm	Particle radius
r_sca	150	nm	Scattering boundary radius
r_pml	200	nm	PML domain radius
$I_{np}$	1	W/cm${}^{2}$	Irradiance power
n_med	1.33	1	Environment refractive index
lambda_in	532	nm	Laser wavelength
a_r	3.5	1	Aspect ratio
l_gnr	2·r0·a_r	nm	Length of NanoRod

## Data Availability

Datasets are available at request.

## Supplementary Materials

The following supporting information can be downloaded at https://www.mdpi.com/article/10.3390/bioengineering10020232/s1. Detail of Theoretical framework. References [10,11,12,13,14,15,16,17] are cited in the Supplementary Materials.

## Abbreviations

The following abbreviations are used in this manuscript:

NPs	Nanoparticle(s)
FDTD	Finite-Difference Time-Domain
FEM	Finite Element Method
LSPR	Localized Surface Plasmon Resonance
MMP	Multiple Multipole Program
MAS	Method of Auxiliary Sources
DDA	Discrete Dipole Approximation
BEM	Boundary Element Method
MBIE	Meshless Boundary Integral Equation
PML	Perfectly Matched Layer
PEC	Perfect Electrical Conductor
PMC	Perfect Magnetic Conductor
