# Supplementary material for: Finite Element Models of Gold Nanoparticles and Their Suspensions for Photothermal Effect Calculation
Source: Bioengineering (Basel). 2023 Feb 9;10(2):232. doi: 10.3390/bioengineering10020232 (PMC9952663; doi:10.3390/bioengineering10020232)
Supplement: Supplementary file 1 [file bioengineering-10-00232-s001.zip › bioengineering-2101479-supplementary.pdf]

Finite Element Models of gold nanoparticles and their suspensions for photothermal effect calculation

J.M. Terrés-Haro, J. Monreal-Trigo, A. Hernández-Montoto, F. J. Ibáñez-Civera, R. Masot-Peris and R. Martínez-Máñez

SUPPORTING MATERIAL

Introduction to plasmonics

The techniques used for the modeling and calculation of the response of a material in an electromagnetic field rely on Maxwell's electromagnetic theory, which is expressed in four partial differential equations for the electric and magnetic fields and describes the behavior of the whole electromagnetic spectrum in classical electromagnetism.

Light interacting with matter may suffer commonly known effects, referred to as interaction with the bulk material. One way to precisely calculate the effect of this interaction in classical physics is to treat the matter as a set of charged particles and study the effect of the electromagnetic field on their position, as the electric and magnetic fields of the incident wave will exert forces on the electrons of the material's atoms, causing them to oscillate [1]. This is the field of plasmonics, which has been developed extensively [2].

It is essential to note that a constant, characteristic of the material being studied, describes this charge density oscillation at an angular speed, which is called the plasma frequency, as indicated in (1).

$$\omega_p^2 = \frac{ne^2}{\varepsilon_0 m} \quad (1)$$

Where the material characteristics are reduced to a number (n) of charged particles (electrons, e) of individual effective mass (m) related to the vacuum permittivity (ε_0). Plugging this into Maxwell's equations, it can be found that the dielectric function of the material (ε) is a function of its plasma frequency, its plasma oscillation damping characteristic frequency (γ) and the incident electromagnetic wave frequency (ω) as expressed in (2).

$$\varepsilon(\omega) = 1 - \frac{\omega_p^2}{\omega^2 + i\gamma\omega} \quad (2)$$

Where (i) is the imaginary operator. This equation is often referred to as the Drude Model, as it makes a simple approach by approximating the metal as a homogeneous domain [3]. Now (2) can be broken down into its real (3) and imaginary (4) parts, where the relation $\tau = 1/\gamma$ is applied.

$$\varepsilon_1(\omega) = 1 - \frac{\omega_p^2\tau^2}{1 + \omega^2\tau^2} \quad (3)$$

$$\varepsilon_2(\omega) = \frac{\omega_p^2\tau^2}{\omega(1 + \omega^2\tau^2)} \quad (4)$$

Each part changes with the properties of the material; normally, at near-visible or visible frequencies, the real part is predominant, but in noble metals, due to interband transitions at those frequencies, ε_2 increases breaking the model in experimental measurements [4], and

absorption predominates over reflection and transmission. In the case of bulk gold, this frequency falls in the blue range, so when illuminated with a white light, the blue is absorbed, and its aspect is yellow [5]. This is the case for bulk materials, where the oscillation is damped and dispersed in their relatively vast volume. However, when nanoparticles come into play, the oscillation is confined to a small volume, and some other effects occur, which will be described in the following lines.

The case of the nanoparticle

When a particle is in an electromagnetic field with a wavelength much greater than its size, a simplification of the analysis can be made by supposing that the phase of the wave that the nanoparticle suffers is the same on each oscillation as represented in Figure 1, so the analysis is made for an electrostatic field. This approximation works well for particles with sizes less than 100 nm.

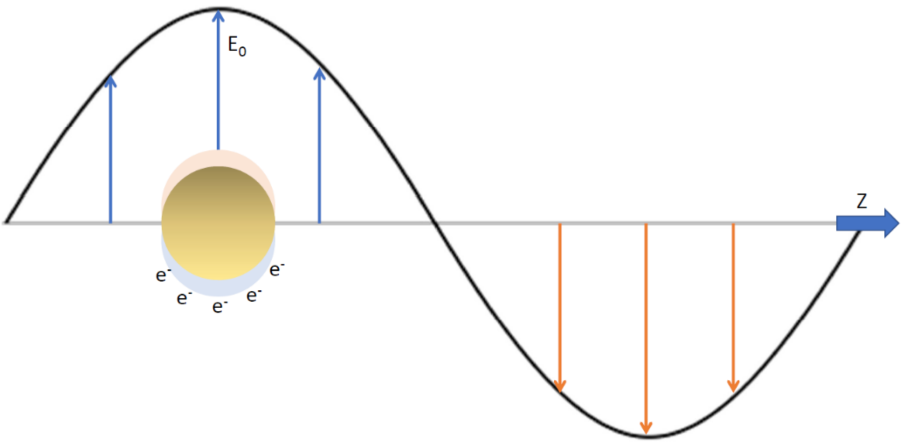

Figure 1. The analysis can be simplified by supposing that a nanoparticle with its dimensions smaller than the wavelength is always affected by the same phase of the electromagnetic wave on its position

Consider a sphere of radius a in a uniform static electric field $E = E_0\hat{z}$, situated in a surrounding non-absorbing medium with dielectric constant ε_m. A solution for the Laplace equation of potential $\nabla^2\Phi = 0$ can be found, from which the electric field $E = -\nabla\Phi$ can also be solved. This problem has a general solution form, once considered azimuthal symmetry, given by (5):

$$\Phi(r,\theta) = \sum_{l=0}^{\infty}\left[A_l r^l + B_l r^{-(l+1)}\right]P_l\big(cos(\theta)\big) \quad (5)$$

Which corresponds to the partial derivative equation solution, giving the series $(A_l r^l + B_l r^{-(l+1)})$ for the r-dependant term and the Legendre Polynomial of $cos(\theta)$ for the θ-dependant term, as the equation takes the Legendre Differential equation form [6].

The sphere is placed in a medium with dielectric constant ε_m, where a homogeneous electric field exists. If the sphere has a different dielectric constant, a charge will be induced in its surface, and the electric field will be distorted. Therefore, the case must be studied for both the electric field outside and inside the sphere, defining (6).

$$E_1 = -\Delta\Phi_1 \quad and \quad E_2 = -\Delta\Phi_2 \quad (6)$$

Where the conditions (7) and (8) apply.

$$\Delta^2\Phi_1 = 0 \quad for \quad (r < a) \quad (7)$$

$$\Delta^2\Phi_2 = 0 \quad for \quad (r > a) \quad (8)$$

At the boundary, the potentials must be equal, so the (9) and (10) relationships must be true for $r = a$.

$$\Phi_1 = \Phi_2 \quad (9)$$

$$\varepsilon \frac{\partial \Phi_1}{\partial r} = \varepsilon_m \frac{\partial \Phi_2}{\partial r} \quad (10)$$

In addition, at a large distance from the sphere, the electric field is still the unperturbed initial field, so (11) applies.

$$\lim_{r\to\infty} \Phi_2 = -E_0 rcos(\phi) = -E_0 z \quad (11)$$

These are the necessary boundary conditions to find the solution coefficients, so the results of the potentials are (12) for the internal potential and (13) for the external potential, which satisfy the partial differential equations and boundary conditions.

$$\Phi_1 = -\frac{3\varepsilon_m}{\varepsilon + 2\varepsilon_m} E_0 r \cos\theta \quad (12)$$

$$\Phi_2 = -E_0 r \cos\theta + \frac{\varepsilon - \varepsilon_m}{\varepsilon + 2\varepsilon_m} E_0 a^3 \frac{\cos\theta}{r^2} \quad (13)$$

It can be observed that (13) describes the superposition of two terms: one is the applied field, and the other corresponds to a dipole located at the particle center that is proportional to the magnitude of E_0 and takes the geometry (by the term radius a^3 into account. From this, the complex polarizability (14) of the spherical particle is extracted.

$$\alpha = 4\pi a^3 \frac{\varepsilon - \varepsilon_m}{\varepsilon + 2\varepsilon_m} \quad (14)$$

Plotting the polarizability against different energy levels applied as an electric field, using different wavelengths, it is found that it suffers a resonant enlargement only when (14) applies [7].

$$\mathrm{R}[\varepsilon(\omega)] = -2\varepsilon_m \quad (14)$$

This states the importance of the environment's dielectric function. When ε_m increases, the resonance wavelength is increased. In addition, the resonant wavelength is affected by the geometry of the particle. When incorporating the solutions of Φ into the distribution of the electric field $E = -\Delta\Phi$ it results in (15) for the inside and (16) for the outside.

$$E_{in} = \frac{3\varepsilon_m}{\varepsilon + 2\varepsilon_m} E_0 \quad (15)$$

$$E_{out} = E_0 + \frac{3\hat{n}(\hat{n}\cdot\vec{p}) - \vec{p}}{4\pi\varepsilon_0\varepsilon_m}\frac{1}{r^3} \quad (16)$$

This implies that, at the exterior of the particle, the electric field is not only its initial value but sums a term that is higher when closer to the particle, so the resonance in the polarizability also

causes an enhancement of the electric field. Because of this, the nanoparticles find a field of applicability in optical devices that rely on sensing changes in the electric field.

Another of the consequences of this resonance is that the particle suffers an enhancement in the efficiency with which it scatters and absorbs light as extensively developed by Bohren and Huffman [8], which resulted in the scattering (17) and absorption (18) cross-section equations for the case of the sphere, as well as for many other symmetric geometries, where $k = 2\pi/\lambda$ and λ is the wavelength of the incident field.

$$C_{sca} = \frac{k^4}{6\pi}\mathrm{R}[\alpha]^2 \quad (17)$$

$$C_{abs} = k\mathrm{I}[\alpha] \quad (18)$$

As the particle's radius is much smaller than the wavelength, absorption dominates over scattering.

[1] Palik, E.D. Handbook of Optical Constants of Solids; Academic Press, 1997.
[2] Maier, S.A. Plasmonics: Fundamentals and Applications; Springer US, 2007. https://doi.org/10.1007/0-387-37825-1.
[3] Fox, M. Optical Properties of Solids; Oxford University Press, 2001.
[4] Johnson, P.B.; Christy, R.W. Optical Constants of the Noble Metals. Physical Review B 1972, 6, 4370–4379. https://doi.org/10.1103/PhysRevB.6.4370
[5] Kolwas, K.; Derkachova, A. Impact of the Interband Transitions in Gold and Silver on the Dynamics of Propagating and Localized Surface Plasmons. Nanomaterials 2020, 10, 1411. https://doi.org/10.3390/nano10071411.
[6] Willets, K.A.; Duyne, R.P.V. Localized Surface Plasmon Resonance Spectroscopy and Sensing. Annual Review of Physical Chemistry 2007, 58, 267–297. https://doi.org/10.1146/annurev.physchem.58.032806.104607
[7] Park, Q.H. Optical antennas and plasmonics. Contemporary Physics 2009, 50, 407–423. https://doi.org/10.1080/00107510902745611.
[8] Bohren, C.F.; Huffman, D.R. Absorption and Scattering of Light by Small Particles; Wiley, 1998. https://doi.org/10.1002/9783527618156.
